# A comparative review of dopant strategies for efficient photo-electrocatalytic water splitting: 3d transition metal oxides vs. carbon-based materials

**DOI:** 10.3389/fchem.2026.1886951

**Published:** 2026-09-14

**Authors:** Mamoona Saeed, Mohsin Javed, Sana Mansoor, Adeeb Shehzad, Sajid Mahmood, Reem Alreshidi, Salah Knani, Shahid Iqbal

**Affiliations:** 1 Department of Chemistry, School of Science, University of Management and Technology, Lahore, Pakistan; 2 Research Center, Dhofar University, Salalah, Oman; 3 Low Dimensional Materials Research Center at Khazar University, Baku, Azerbaijan; 4 Department of Physics, College of Science, Northern Border University, Arar, Saudi Arabia; 5 Center for Scientific Research and Entrepreneurship, Northern Border University, Arar, Saudi Arabia; 6 Department of Chemical and Environmental Engineering, University of Nottingham Ningbo China, Ningbo, China

**Keywords:** carbon-based materials, quantum dots, solar energy, transition metal oxides, water splitting

## Abstract

Photocatalytic water splitting is an essential technology in the production of hydrogen on a sustainable basis, to combat the energy limits the world is facing by conserving solar energy in chemical bonds. Nevertheless, an inefficient visible light absorption and recombination of charges usually restrict the efficiency of semiconductor photocatalysts. This review is comparative analysis of two prominent dopant-based methods to overcome these limitations, namely, Transition Metal Oxides (TMOs) and Carbon-Based Materials (CBMs). We debate on the latest advancements in TMO dopants including Sc, V, Cr, Co, Ni and Cu, which is a TMO dopant but acts by decreasing bandgaps and altering electronic band structures in order to reach visible light. On the other hand, CBMs as graphene, carbon nanotubes (CNTs), and carbon quantum dots (CQDs), are electron sinks, as well as, conductive scaffolds, which enable separation of charge, and charge movement. This review emphasizes the trade-off between TMO-based bandgap engineering and CBM-based charge-mobility optimization. Additionally, we are referring to the combination of these strategies into hybrid systems that combine the optical benefits of TMOs and kinetic benefits of CBMs. The current review gives a design rulebook to the next-generation of high-efficiency photocatalysts in that it outlines some of the special conditions under which each type of dopant can work effectively**.**

## Graphical Abstract

**Figure:**
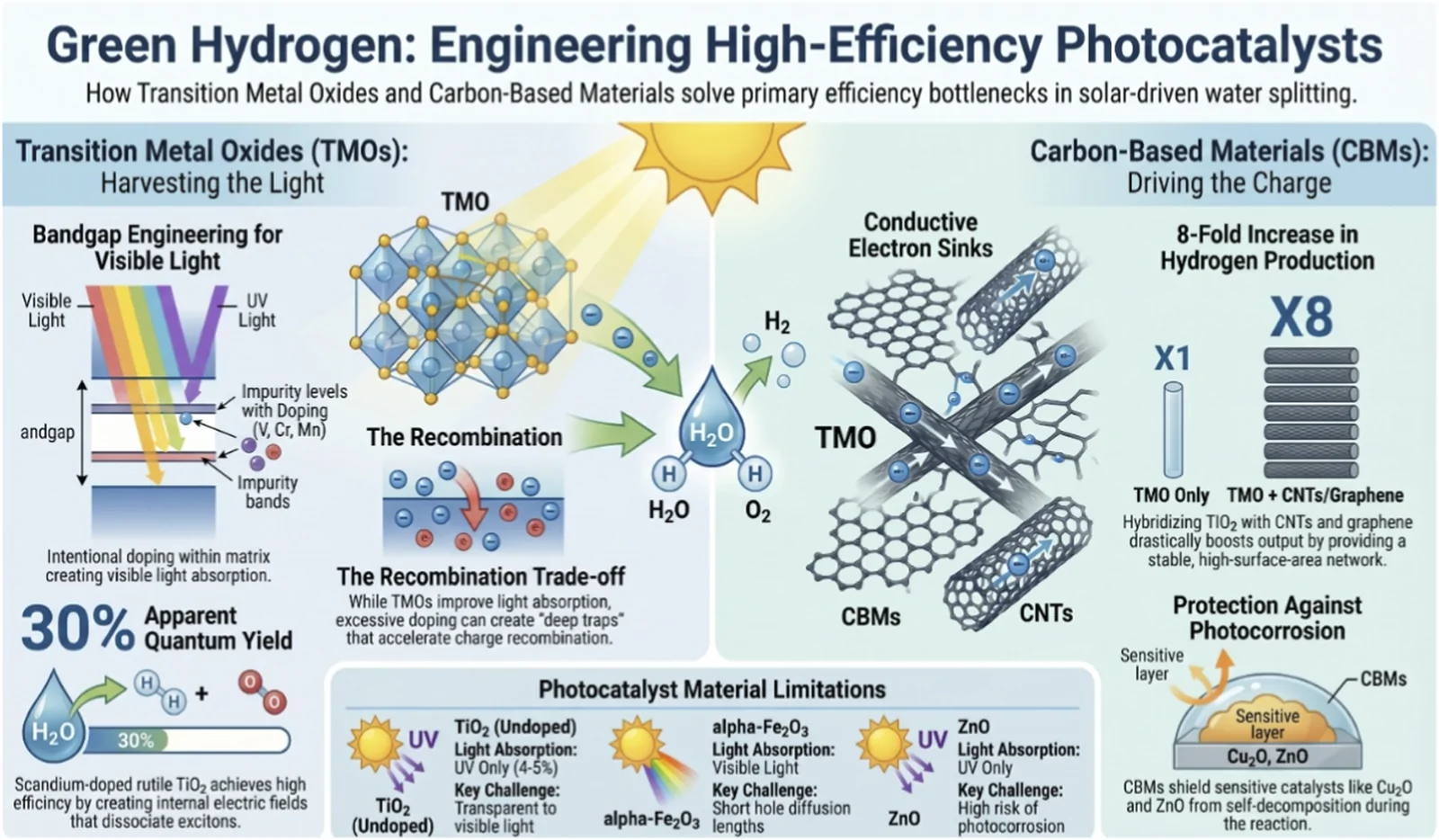

## 1 Introduction

The escalation of global climate change has led to an immediate transition of fossil fuel use to renewable energy carriers, and hydrogen (H_2_) has been the buzzword as the solution to a sustainable energy future. To address the intermittency challenges that can be posed by the generation of solar power, photocatalytic water splitting to the production of green hydrogen can be an attractive way to store solar energy in chemical bonds. Due to the increased urgency of the new global climatic crisis and the Net Zero by 2050 targets, the necessity to motivate this response increased. Hydrogen generated through this process can be used as a carbon-neutral source of energy, which used to decarbonize traditionally hard-to-abate industries, including heavy industry and long-haul transport, as an alternative to fossil fuels. The production of this hydrogen is however very critical since despite the current technology that is the steam methane reforming, it produces a lot of CO_2_ and therefore innovation of clean methods of production such as water splitting is an international issue (Acar et al., 2016; Armijo et al., 2022).

A thermochemical reaction is the splitting of the molecules of water (H_2_O) to form its two elements, hydrogen (H_2_) and oxygen (O_2_). Water splitting is a thermodynamically uphill reaction because water is very stable and its decomposition requires an external energy input. Under standard conditions, the minimum Gibbs free energy that must be supplied is approximately 237 kJ per mole of water, which translates to a theoretical minimum voltage of 1.23 V. The general water-splitting reaction expressed as the following stoichiometric reaction:

$$\begin{array}{cc} 2H_{2}O\;\left(l\right)\;\to 2H_{2}\;\left(g\right)+O_{2}\;\left(g\right) & \Delta G^{\circ }=+237\text{ kJ}/\text{mol} \end{array}$$

Oxidation evolution reaction (OER): This is the oxidation half-reaction, which is taking place at the valence band, where holes oxidize water to form oxygen and protons.

$$2H_{2}O\;\left(l\right)+4h+\to \;O_{2}\;\left(g\right)+4H^{+}$$

Hydrogen Evolution Reaction (HER): This reduction half-reaction occurs at the conduction band (CB) where photo-excited electrons reduce protons to hydrogen gas.

$$4H^{+}+4e-\to \;2H_{2}\;\left(g\right)$$

### 1.1 Methods of water splitting

There are three main thermochemical directions commonly investigated to obtain the water splitting: thermolysis, electrolysis and photocatalytic (or photoelectrochemical) splitting. Thermolysis is the process of heating water to an extremely high temperature (>2500/C) and is energy-intensive, materialistically dissenting, which makes it the least efficient. Electrolysis is a well-established, high efficiency technology that employs electricity to divide water but the environmental impact of this process is completely driven by the origin of that electricity; when the power grid is composed of fossil fuels, the resulting hydrogen is not green. Conversely, photocatalytic water splitting is the most promising long-term solution that has been widely discussed in contemporary literature since it incorporates the harvesting of solar energy with the fuel storage into a direct process (Chen et al., 2017; Takanabe, 2017).

### 1.2 Mechanism of photocatalytic water splitting

**FIGURE 1:**
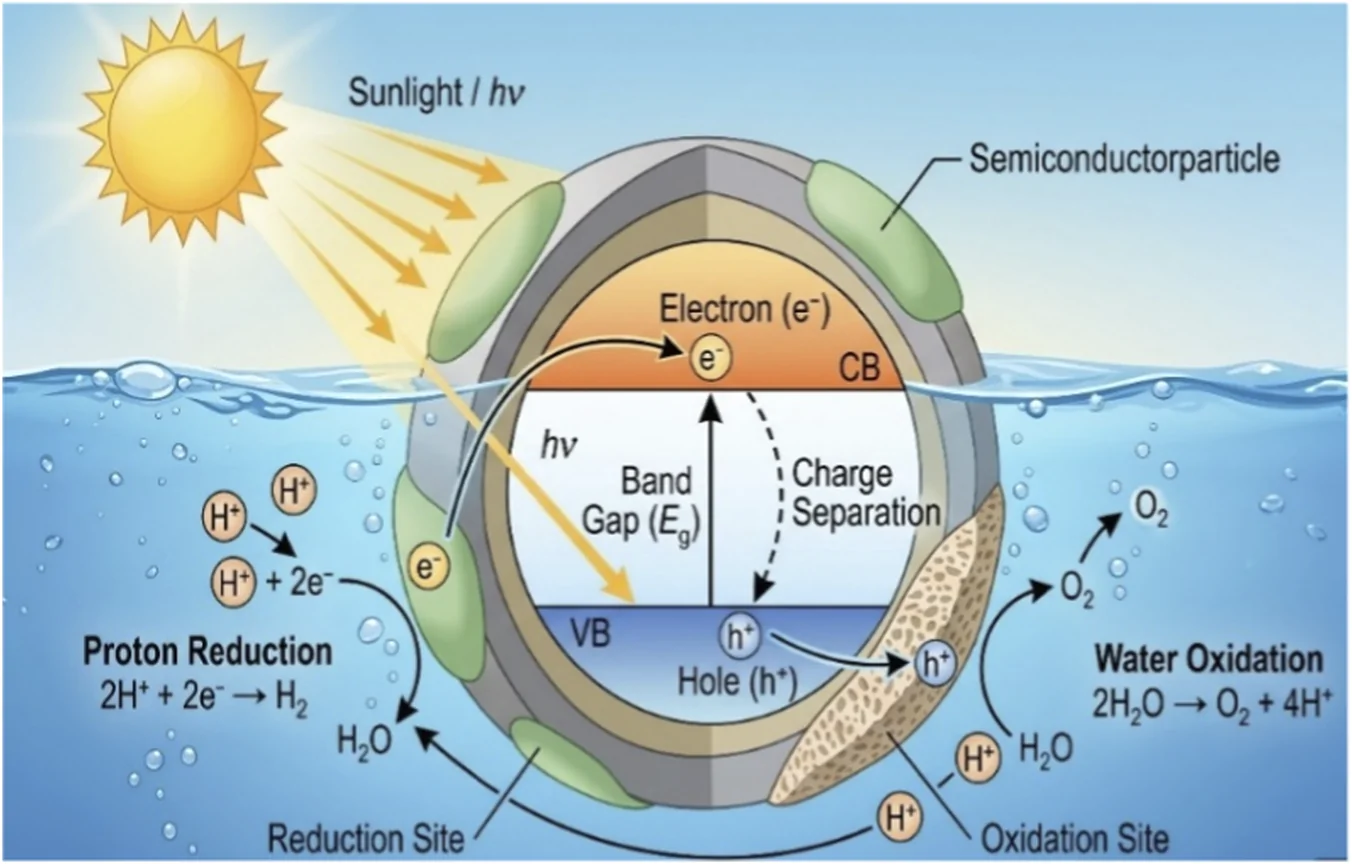
Schematic illustration of photocatalytic water splitting. This figure was generated by the authors using Genspark and Notebook, and subsequently edited and annotated using Adobe Illustrator.

As the photocatalytic reaction utilizes the photo-excited electrons (e^−^) and holes (h^+^) migrating to the surface of the photocatalyst, a second important requirement is that the photocatalysts should exhibit efficient separation and migration of the photo-excited carriers (Figure 1). As photo catalysis uses water and photon energy, it does not produce any pollutants. Subsequently, it will contribute substantially to the overall solution of energy and environmental problems in the nearest future. The main role of a photocatalyst is to generate charge carriers, i.e., electrons and holes, by absorbing incident light.

1. Absorption of light with wavelengths similar to the band gap of the semiconductor causes electrons in the valence band (VB) to be pulled out of it to the conduction band (CB), leaving holes in the valence bond (VB).
2. The second (2) step is the separation and migration of the electron-hole pairs that were created during the photo generation process. Ideally, they ought to be all the electrons and holes to the surface and do not recombine and this renders the photocatalyst as effective as possible. In the final step, the electrons that depart the conduction band (CB) and arrive at the surface of the catalyst reduced and form hydrogen, and holes that depart the valence band (VB) and arrive at the surface of the photocatalyst oxidized and become oxygen. Higher redox potential, effective light absorption and photo induced charge prevention recombination are convenient chemical conversion methods that enhance productivity of photocatalytic process (Ali et al., 2024; Eidsvåg et al., 2021).

The electronics band structure of the semiconductor materials determines the mechanism of photocatalytic water splitting. On absorbing a photon having more energy than the bandgap, an electron is raised out of the valence band (VB) into the conduction band (CB), leaving a hole in the former. In order to effect the reaction in an effective way, the reduction potential of H^+^/H_2_ must be more negative at the bottom of the conduction band (CB) than the oxidation potential of O_2_/H_2_O^+^ (1.23 V vs. NHE) at the top of the valence band (VB) to induce the Oxygen Evolution Reaction (OER) and, conversely, the Hydrogen Evolution Reaction (HER). Such reduction and oxidation reactions necessitate that the electrons and holes generated on the photo surface at the time must migrate to the catalyst surface without recombining to generate such two reactions respectively (Hisatomi et al., 2014). Solar photocatalytic water splitting needs photocatalyst which is a photosensitizer since water is not visible. The optimal material should be one that has an appropriate band gap and band alignment to redox reactions, be chemically stable and corrosion resistant. It should also be cost effective in the sense that the cost should be low and in large quantity, recyclable and able to be produced in large quantities (Acar et al., 2016).

Fujishima and Honda reported the first report on heterogeneous photocatalytic hydrogen production by water splitting on a TiO_2_ semiconductor electrode in 1972, laying the groundwork for semiconductor-assisted solar fuel generation. The photocatalytic hydrogen production system is heterogeneous, in which the semiconductor used as the light harvester as well as a catalytic platform for generation and interfacial redox reactions. Lehn and co-workers were the first to report one of the earliest homogeneous photocatalytic hydrogen production systems in 1977 based on the use of triethanolamine as a sacrificial component in a molecularly mediated photo redox process. In typical homogeneous photocatalytic hydrogen production systems, an organometallic catalyst used in conjunction with a molecular photosensitizer/electron donor, which allows light harvesting to be decoupled from catalytic turnover. More recently, hybrid photocatalytic systems have been developed, including the combination of features from both heterogeneous and homogeneous platforms, which involve the use of semiconductor photocatalysts in combination with molecular catalysts and photosensitizers that enhance charge separation and catalytic efficiency (Corredor et al., 2019; Fujushima, 1972; Kamat and Sivula, 2022).

### 1.3 Heterojunction and types of heterojunctions on basis of charge transfer

The interface created by two semiconductors with different band structures known as a heterojunction, and it produces certain band alignments that control charge transfer behavior.

#### 1.3.1 Type I heterojunction

In Type I (straddling) configuration, the conduction band (CB) and valence band (VB) of a smaller bandgap semiconductor are contained in the larger bandgap semiconductor bandgap. At the opportunity of light irradiation, the charge carrier generated by the light is transported to the smaller bandgap material by the larger bandgap part, and the charge is concentrated in the smaller bandgap material. Although this arrangement may be better than the individual components in case the materials are carefully selected, considered having low charge separation efficiency since they all electrons and holes are concentrated in the same semiconductor, and thus they recombine.

#### 1.3.2 Type II heterojunction

In a Type II (staggered) structure, the conduction and valence level of one semiconductor is greater than that of its counterpart. This structure compels the electrons and holes to move in opposite directions thus the charge carriers are effectively separated. Therefore, Type II heterojunctions expected to be highly photocatalytic since they have a better proficiency in charge separation. Nevertheless, this mechanism has a serious thermodynamic limitation: the enhanced separation is obtained to the cost of decreased redox potential. As the carriers shift to lower energies, the electrons deprived of reduction power and the holes become deprived of oxidation power weakening the driving force of the chemical reactions (Balapure et al., 2024; Low et al., 2017a).

#### 1.3.3 Z-scheme

**FIGURE 2:**
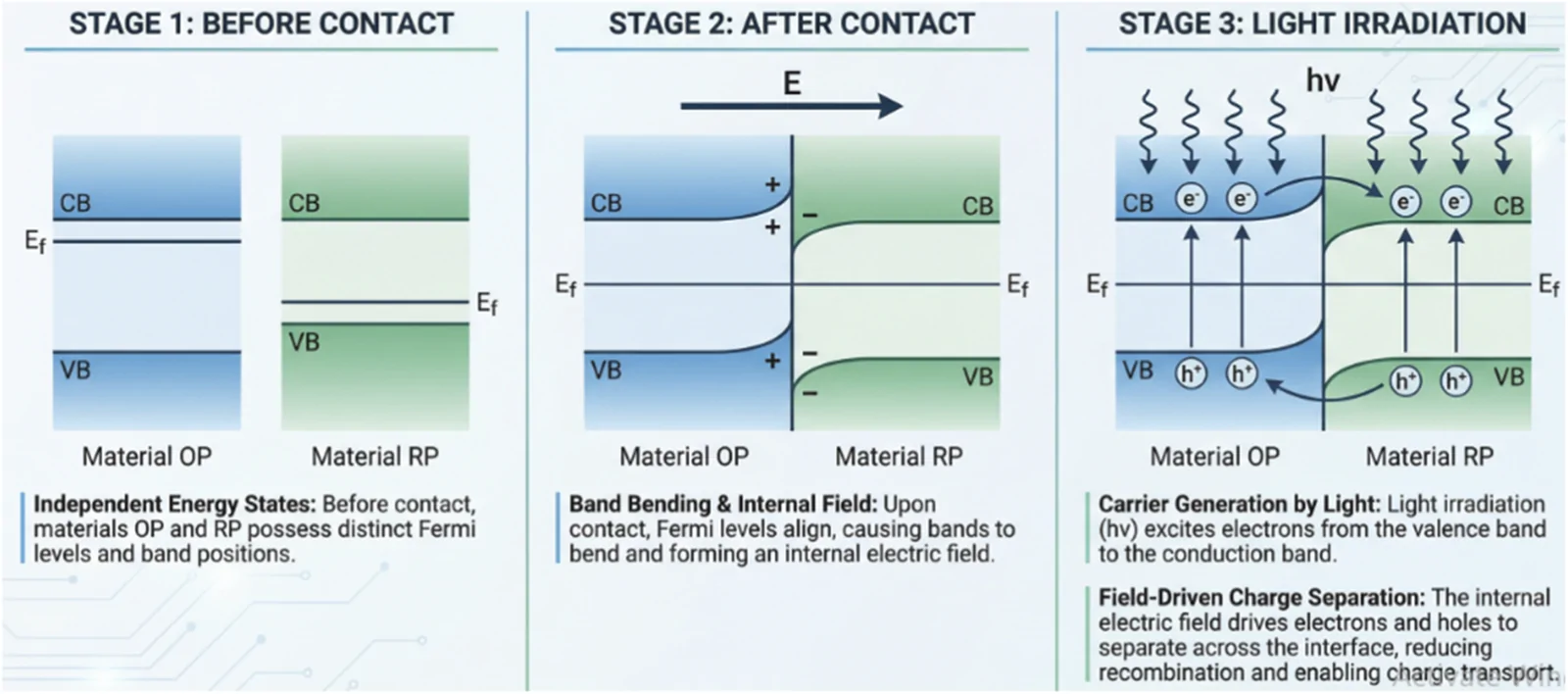
Schematic illustration of Z-scheme heterojunction. This figure was generated by the authors using Genspark and NotebookLM, and subsequently edited and annotated using Adobe Illustrator.

Despite the structural similarity of the direct Z-scheme photocatalyst with the Type-II heterojunction, the photocatalyst follows a different pathway in charge carrier migration, which is similar to the letter Z (Figure 2). The electrons produced by the photo-generated surface of Semiconductor B will have lower reduction ability than the electrons produced by the photo-generated surface of Semiconductor A and the holes produced by the photo-generated surface of Semiconductor A will have lower oxidation ability than the electrons produced by the surface. It holds the electrons of high reduction potential in Semiconductor A and the holes of high oxidation potential in Semiconductor B, which also keeps the total redox potential of the system at the optimum value. In addition, the physical aspect of this makes it more viable to charge the migration than Type-II heterojunctions because this is attributed to the electrostatic attraction between the recombining electrons and the holes (Low et al., 2017b). The heterojunctions of type II increase the efficiency of charge separation and promote the movement of electrons and holes in opposite kinetic directions. Z-scheme systems. This traditional system is a replica of the photosynthesis mechanism, where mediators used to recombine weak charges without loss of high redox potential. The direct Z-scheme removes these mediators, and they are similar to Type II structures except that they have a Z-shaped transfer pathway. This is a mediator-free method to prevent the backwards reactions as well as prevent the shielding effects of the mediated systems (Jiang et al., 2018).

#### 1.3.4 S-scheme

**FIGURE 3:**
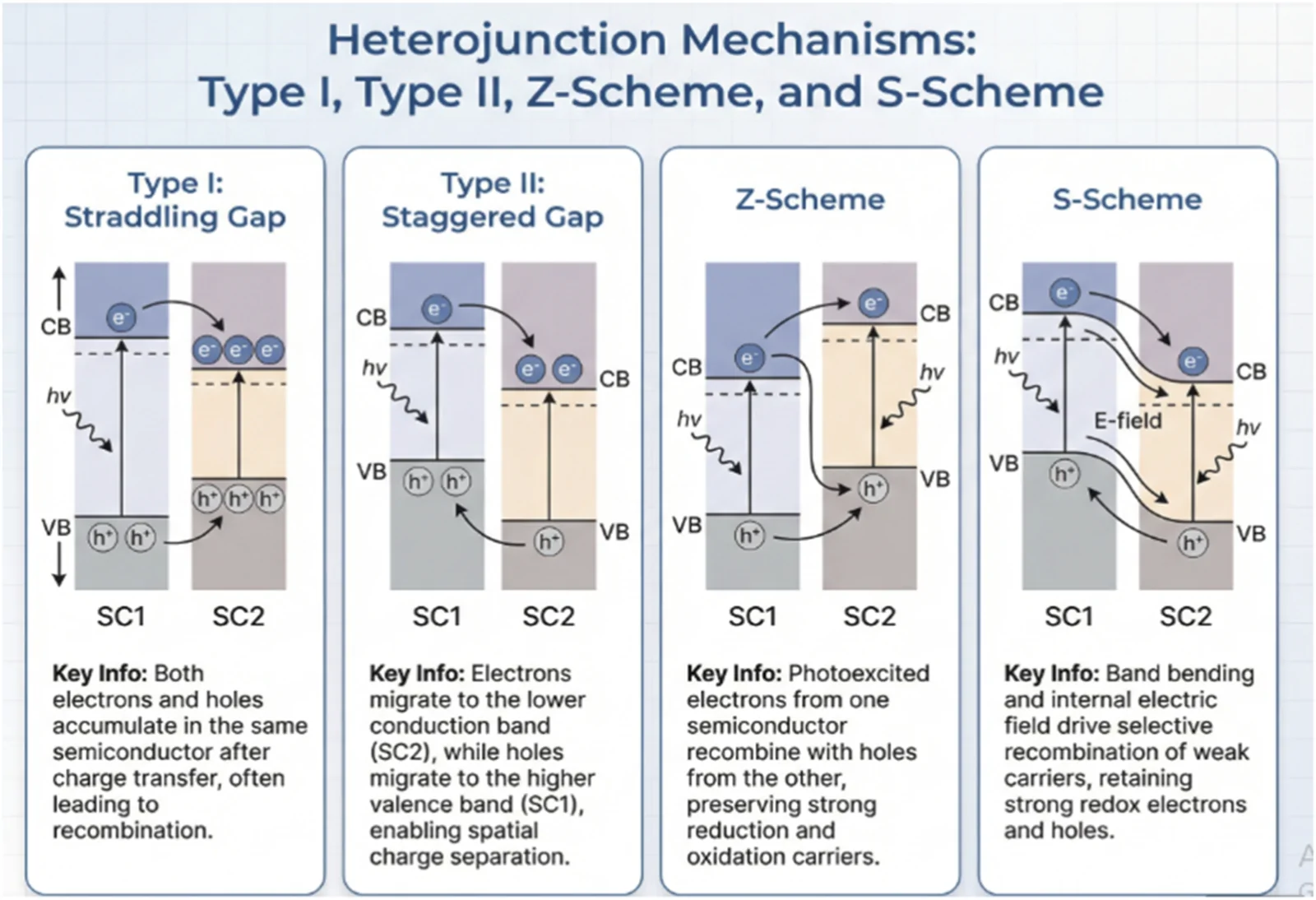
Energy band diagrams and charge transfer mechanisms for all semiconductor heterojunctions. This figure was generated by the authors using Genspark and NotebookLM, and subsequently edited and annotated using Adobe Illustrator.

The S-scheme heterojunction was created to address the limitations of Type-II and Z-scheme systems with the assembly of an oxidation and a reduction photocatalyst (Figure 3). At contact, the flow of electrons forms an intrinsic electric field and band bending which provides the recombination process of weak charge carriers. This process retains the strong electrons and holes, has high redox potential, and can be used to effectively separate charges. As a result, S-scheme is an improved photocatalyst with optimal performance in terms of charge separation and reaction driving force (Zhang L. et al., 2022).

Because of their outstanding stability and empty d orbit structure, TMOs are frequently employed in a variety of applications, including thermal catalysis, photo catalysis, and electro catalysis. However, morphological engineering, crystal surface engineering, and oxygen vacancy engineering were used to enhance the catalytic performance of TMOs due to their low catalytic activity and selectivity. The main structure of most photocatalysts consists of a semiconductor material with other cocatalysts added as required. Semiconductors can absorb high-energy photons that are beyond their bandgap, the difference in energy between the valence and conduction bands (E_bg_ = E_CB_ -E_VB_), and can convert them into chemical or electrical energy. Cocatalysts of transition metals can be classified as either electron-trapped reduction cocatalysts to photocatalytic H_2_ evolution or hole-trapped oxidation catalyst to photocatalytic O_2_ evolution depending on the types of trapped charge carriers and their roles in photocatalytic water splitting (Zhao et al., 2022). As a result, the use of 3d-metal clusters as photocatalysts earned a lot of attention in recent years. Co, Cu, Mn, Ni, and Ti are only a few of the transition metal clusters that have been employed thus far for photocatalytic oxygen or hydrogen synthesis. In addition to activating the metal clusters for water splitting, altering these materials’ structures (metal decorating, ligand regulation, or nuclearity) can also promote charge separation and increase the materials’ stability under photoexcitation (Chen et al., 2020). True dopants are incorporated into the host lattice and modify the electronic band structure of the semiconductor, while transition-metal oxide cocatalysts including Fe_2_O_3_, Co_3_O_4_, and NiO have also been extensively investigated to enhance photocatalytic performance by offering surface active sites, promoting interfacial charge transfer, and suppressing electron–hole recombination (Pozan et al., 2013).

Materials of carbonaceous origin are currently being used to promote the photocatalytic activity of semiconductors. In addition to preventing recombination of charges, the presence of carbon nanostructures inhibits recombination and produces a hydrophobic microenvironment that is useful in concentrating reactants around the active sites (Ng et al., 2012). Carbon materials provide the best support because they may contribute significantly to the enhancement of CO_2_ adsorption and activation (Sundar et al., 2023). The collaboration of photocatalyst and carbon materials improves product yields by large margins because of the considerable surface area and large-scale conjugation of the electrical aspects of the products, the use of widespread process of the electrons circulation. These special surface properties make carbon materials very good conducting substrates of photocatalysts. As a result, they can be combined as a viable approach to realization of improved CO_2_ reduction through photo catalysis (Kandy, 2020). The use of carbon-based materials has great benefits in the heterogeneous catalysis mode that includes a high degree of chemical and thermal stability in acidic and basic conditions, low levels of corrosion and hydrophobic properties. Moreover, they offer convenient advantages like ready access out of reaction mixtures and affordability (Khan, 2021).

### 1.4 Mechanistic basis of carbon-based materials and transition-metal-oxide dopants

For transition metal oxide dopants, the mechanism should be explained more. Such dopants incorporated into the crystal lattice of the host material and modify its electronic structure in two ways: via d-orbital interactions and by the creation of impurity-states. The energy levels of d-electrons are affected by the crystal field formed by the surrounding atoms, which may split the d levels and produce new energy levels close or even inside the energy gap. These states can absorb visible light if the position is suitable and can trap electron and hole and accelerate the electron–hole recombination if the position is too deep. However, in the case of some dopants, like Mn and Cu, the local distortion can further impact the structure and charge transport, and it is important to do density-of-states analysis to determine whether the dopant enhances or degrades the photocatalytic activity (Krishnan et al., 2024; Mishra et al., 2025).

In the case of carbon-based materials, the main point is that graphene, carbon dots, and carbon nanotubes can help to transport electrons away from the semiconductor surface. Recent review studies reveal that photocatalytic properties of carbon-based materials are better due to its conductivity, adsorption property and charge transfer phenomenon at the interface. The distribution of electrons between two materials is driven toward a balance in energy levels, resulting in a natural creation of a Schottky-type interface that enhances forward electron transfer and reduces back transfer. The carbon material, in its simple terms, acts as an electron partner that keeps the electron generated by light “available” for reaction, for example, to produce hydrogen. Thus, the term active interfacial charge-management component is more appropriate than the term passive supports for carbon-based materials (Ahmed and Nishina, 2026; Syed et al., 2019).

There are many reviews on photocatalytic water splitting, yet most focus on a single aspect: transition metal doping, carbon doping, heterojunction construction, loading of cocatalysts and/or defect engineering. This makes it difficult to find a concise comparative analysis that elucidates the mechanistic roles and performance benefits and limitations of TMO-based dopants versus CBMs, making the field less accessible for newcomers. This review will critically discuss the two types of modification strategy with respect to band-structure tuning, defect chemistry, charge separation, interfacial transport, and catalytic surface reactions. The study intends to unify the literature in the same mechanistic framework for a clearer design principle in selecting and combining the different dopant approaches for more efficient photocatalytic water splitting.

As shown in Table 1, the existing literature divided into individual discussions of TMOs, transition-metal-based cocatalysts, carbon-based materials, and general hydrogen-production systems, whereas the current review offers a comparative and mechanism-driven framework to evaluate TMO-based dopants and CBM-based strategies in photocatalytic water splitting. For this review, the literature was systematically searched in Scopus, Web of Science, Science Direct, Wiley Online Library, Springer Link, and Google Scholar with the combinations of the keywords: photocatalytic water splitting, photoelectrochemical water splitting, transition metal oxide doping, carbon-based materials, heterojunction, Type II, Z-scheme, S-scheme, hydrogen evolution, and defect engineering. All publications in English language (articles, reviews, primary research papers) were included in the search, but only for the period relevant to this topic. Studies accepted if a clear mechanistic discussion was provided, quantitative photocatalytic performance data provided, or material systems, which were directly relevant to the study. The studies were excluded if they were duplicate, insufficient experimental or mechanistic information, editorials, conference abstracts and outside of the scope of photocatalytic water splitting. Relevant and quality articles were selected by screening the title and abstract first, and then performing a full text assessment.

**TABLE 1:** Comparative overview of representative prior reviews on transition metal oxides, carbon-based materials, and photocatalytic hydrogen evolution.

Materials covered	Application focus	Main gap addressed by present review	References
Transition metal oxides	Photo catalytic dye degradation	Does not compare TMO strategies with carbon-based materials for water splitting	Krishnan et al. (2024)
Transition-metal-based cocatalysts	Photo catalytic water splitting	Focuses on cocatalysts, not lattice dopants or CBMs	Zhao et al. (2022)
Carbon-based catalysts	Photocatalytic and photoelectrochemical water splitting	Discusses carbon materials alone, without direct comparison to TMO-based strategies	Lim et al. (2019)
Transition metal oxides	General photo catalysis	Does not provide a side-by-side mechanistic framework with carbon-based modifiers	Imtiyaz and Singh (2024)
General photocatalytic hydrogen production systems	Heterogeneous, homogeneous, and hybrid photo catalytic systems	Offers a wide overview of hydrogen generation, but does not specifically compare TMO-based and carbon-based modification strategies in the context of photocatalytic water splitting	Corredor et al. (2019)

## 2 Discussion

### 2.1 Undoped transition metal oxides in photocatalytic splitting of water

Pure Scandium oxide Sc_2_O_3_ Bear a bandgap between 5.6 eV and 6.0 eV. This locates it in the Ultraviolet (UV) region. Given that the solar spectrum arriving on the surface of the earth is extremely low in energy below an approximate of 300 nm, Sc_2_O_3_ is a good insulator/transparent window in the sunlight. It is unable to produce the charge carriers (electrons and holes) to catalyze the water-splitting reaction**.** The trivalent oxide Sc_2_O_3_ with large bandgap (E.g., = ∼6 eV), high dielectric constant (ɛ = ∼14), and suitable band offset (BO), has been reported to deliver low density of trap states and little leakage current at the Sc_2_O_3_/GaN interface in the past decade (Zhang et al., 2019).

The Scandium ion (Sc^3+^) has an empty d-shell (d^0^), resulting in very low electronic polarization and poor charge mobility even if carriers were generated.

The photocatalytic mechanism of vanadium pentoxide (V_2_O_5_) starts with the absorption of photons that excite electrons from the valence band (VB) to the conduction band (CB), forming electron-hole pairs that drive surface reduction and oxidation (redox) processes.

However, the fast recombination of this photon generated charge carriers, which is enhanced because the small band gap and results in waste of the energy as heat or light emission, makes the application of the pure V_2_O_5_ difficult. This issue has been addressed by doping methods involving transition metals (such as Co., Fe, Ni, and Cu) and non-metals. These dopants create intermediate energy levels, which act as effective electron traps in the band gap, extend the lifetime of the charge carriers and reduce recombination (Li et al., 2023; Tallal et al., 2026). According to band structure studies, certain phases of vanadium oxide have conduction band (CB) edges that are more negative than the hydrogen reduction potential, indicating that they are thermodynamically capable of evolving hydrogen. The order of photocatalytic performance under visible light irradiation was VO_2_ <V_2_O_5_<VO _2_ (M) <V_6_ O_13_, which is consistent with their respective energy band locations. All materials have appropriate band gaps to efficiently drive visible-light photo catalysis, according to UV-vis spectroscopy, which showed absorption edges ranging from 397 to 573 nm that were attributed to d-d transitions and ligand-to-metal charge transfers (Shen et al., 2012).

Manganese oxides, such as MnO, Mn_2_O_3_ and MnO_2_ look dark seem to absorb light very well.

However, they are not hosts because they do not transport charge well and are not chemically stable. Manganese oxides have something called valency, which means they have different charges, like Mn^3+^ and Mn^4+^. When charge transport happens, it is through a process called “polaron hopping” where electrons jump from one ion to another. This process is much slower than the way charge transport works in materials like TiO_2_. As a result, when light hits oxides the excited electrons and holes combine again before they can help split water (Robinson et al., 2013).

One big problem with oxides is that they dissolve easily in water. This is because of something called the Jahn-Teller distortion, which happens with Mn^3+^ ions and makes the structure of the material weak. This is especially true in conditions, which are common when trying to split water or even in neutral conditions over a long time. Manganese oxides are used for cleaning up pollutants or helping with the Oxygen Evolution Reaction. However, manganese oxides not used as the material, for absorbing light to produce hydrogen efficiently because they produce very little electric current and break down quickly. Manganese oxides just do not work well for this purpose (Wang et al., 2023).Chromium (III) oxide, known as Cr_2_O_3_ is not a good semiconductor. Chromium (III) oxide has a gap between its energy levels about 3.4 eV. This means Chromium (III) oxide only absorbs light, which is a big limitation. There are also some issues with using Chromium (III) oxide. For example, when we try to split water using Chromium (III) oxide it can become toxic. This happens because Chromium (III) can turn into Chromium (VI), very bad for the environment. This is not bad for the environment but it also means that Chromium (III) oxide does not work well as a catalyst. When light hits Chromium (III) oxide, it absorbs that light. However, this absorption is mostly due to the way the electrons move around the Chromium (III) oxide molecule. These movements are called d-d transitions. The problem with these transitions is that they do not create free electrons that can move to the surface of the Chromium (III) oxide. This means that Chromium (III) oxide is not very good, at using light to split water (Kasikov et al., 2019).

The decades of studies investigating the characteristics of hematite have found some drawbacks that do not allow obtaining high solar light harvesting efficiencies. Nevertheless, the combination of the desirable and favorable characteristics of hematite (i.e., bandgap of 2.0 eV, unprecedented stability, and abundance) render hematite an evident and clear solution to solar energy storage, through photoelectrochemical water splitting. Here we have observed how the optoelectronic characteristics present a paradox, which can be managed through a careful control of substitutional doping and morphology. Together with certain methods to mitigate electrochemical losses at the interface, these Nano structuring methods have demonstrated an incredibly fast rate of photocurrent on hematite at standard conditions (Sivula et al., 2011).

One of the key issues in the development of β -Fe_2_O_3_ to be used in photoelectrochemical is the challenge of its thermodynamic metastability. This sensitivity is particularly emphasized by the fact that this phase is prone to transforming into a different phase when characterizing the optical properties. It has been reported that standard Raman spectroscopy with either 532 nm or 633 nm laser can induce a localized conversion of β **-** Fe_2_O_3_ to the thermodynamically stable α Fe_2_O_3_. This energy conversion under laser light demonstrates the fine balance of energy in the metastable state and the need to use longer wavelengths, e.g., 785 nm, to verify the structure accurately without destroying the specimen. Although this high-intensity local heating is an extreme case, it highlights the significance of phase stabilization measures like Zr-doping, to make sure that the material is in the active beta state when it is in use (Zhang et al., 2020).

**FIGURE 4:**
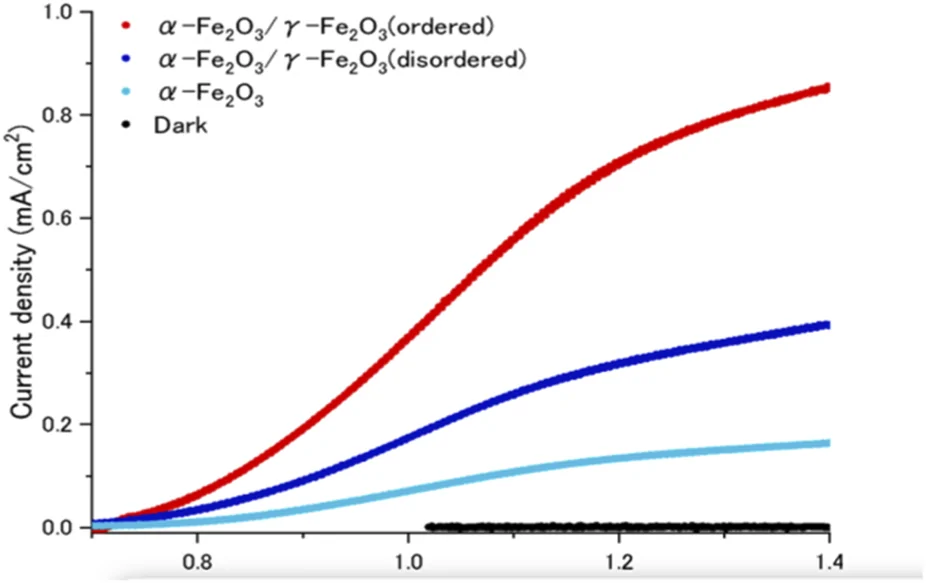
Photocurrent density-potential (I–V) curves of α-Fe_2_O_3_ (light blue), α-Fe_2_O_3_/γ-Fe_2_O_3_ (disordered) (blue), α-Fe_2_O_3_/γ-Fe_2_O_3_ (ordered) (red) and dark current (black) (Tokubuchi et al., 2021).

The combination of a magnetite (γ-Fe_2_O_3_) interlayer between the hematite (α- Fe_2_O_3_) photo anode and FTO substrate is a major improvement to photoelectrochemical (PEC) operation. At 1.23 V, the photocurrent density of 0.74 mA/cm^2^ (ordered) was obtained when using α - Fe_2_O_3_/γ - Fe_2_O_3_ composite, much higher than bare α-Fe_2_O_3_ (0.14 mA/cm^2^). Time-resolved spectroscopic analysis (HD-TG) indicated that this is due to an increase in charge separation efficiency. The insertion of particles of γ- Fe_2_O_3_ helps in the transfer of photo-excited electrons in α- Fe_2_O_3_ layer and hence inhibits the recombination. In particular, it was found that the recombination rate of surface-trapped holes in the composite was two times lower than that of pristine hematite, which validates the point that the recombination of surface-trapped holes takes place two times more slowly in the composite. The interlayer of Fe_2_O_3_ is vital in enhancing the lifespan of charge carriers in order to divide water effectively (Figure 4; Tokubuchi et al., 2021).

The overall water splitting (POWS) reaction can proceed on TiO_2_ **-**based photocatalysts of varying phases (anatase), rutile and brookite) at the long-term UV light irradiation. We have discovered that the stable overall water separation at the stoichiometric ratio of H_2_/O_2_ is possible on rutile TiO_2_. There are numerous trapped states beneath the Fermi levels of anatase and brookite TiO_2_ (no trapped states are present at rutile TiO_2_), which has the potential of lowering the over potential of water oxidation thermodynamically (Li et al., 2015). In contrast to anatase and rutile, brookite TiO_2_ possesses a reasonable depth of the electron trap that can facilitate both photocatalytic oxidations and reduction processes, despite the fact that brookite TiO_2_ is typically thought of as a photo catalytically less active material (Cao et al., 2020).

Undoped TiO_2_ is the standard material in photocatalytic water splitting, as it is highly chemically stable, non-toxic, and with appropriate band edges distribution, which crosses the water redox potentials. Its impact is however limited to ultraviolet (UV) (bandgap of 3.05–3.2 eV) that is limited to a factor of 4–5 percent of the solar spectrum. While undoped TiO_2_ transports charges excellently when excited and its recombination is low, but its failure to make use of visible light decreases its general solar-to-hydrogen efficiency. It serves as a paradigm system to learn about charge transfer kinetics (Ni et al., 2007). The cuprous oxide is prone to degradation in water in the presence of light since by intra band gap corrosion reactions; therefore, this is overcome by placing protective over layers and co-catalysts to help stabilize the oxide and reduce the over potential of hydrogen evolution (Tilley, 2023).

A p-type semiconductor with a broad bandgap (>2.6 eV), cobalt oxide (CoO) gained a lot of attention because of its optical and electroactive characteristics. Bao et al. made an important discovery when they showed that CoO nanoparticles could photo catalytically break down water under visible light with around 5% efficiency without the need for sacrificial reagents or co-catalysts. One-dimensional (1D) nanostructures, such as nanobelts, nanowires, or nanorods, are used in place of nanoparticles to further improve performance. These 1D structures reduce ion diffusion lengths and offer a direct charge transport channel without crystal boundary resistance when they crystallize in a rock salt structure. When compared to systems based on nanoparticles, their greater surface area also helps to increase the electrolyte-electrode contact zone, which results in better rate properties and photocatalytic activity (Liu et al., 2017).

The yield of hydrogen of Co_3_O_4_ is three times that of Co_3_O_4_.CoO and CoO. These findings are consistent with the photocurrent density findings. A number of aspects are involved in the photocurrent density and hydrogen generation, including morphology and surface area. The primary reason that Co_3_O_4_ had a high photocurrent density than the others was its large surface area. The nanocubic CoO photocatalysts were the least active of all the samples because the primary particles aggregated and the active surface of the active species with which the reactants can interact was low. The CoO mixture phase was characterized by a low photocurrent density than Co_3_O_4_ due to the nanocubic shape of the CoO particles (Guo et al., 2022). The photocurrent density is often reported to be higher in the case of Co_3_O_4_, but this is not only due to the bulk phase. The morphology such as particle size, porosity, exposed crystal facets and interparticle connectivity plays an important role in its performance. Nanostructured Co_3_O_4_ can offer a greater fraction of accessible active sites and shorter charge transport pathways, which will reduce recombination and enhance the total photocatalytic response. It should therefore not be interpreted as a phase-only advantage in some of the studies that show the superiority of Co_3_O_4_ over CoO as this is a morphology-dependent effect (Subagyo et al., 2023). The findings of the research show that Cobalt Oxide Co_3_O_4_ contains great potential as a photocatalyst in the hydrogen generation and that its applications are not limited to thermochemical reactions. The most important aspect of optimizing such a performance is the size of the particles, or rather the nano crystallinity; smaller particles have higher surface area but this has to be balanced to maintain crystallinity since a further reduction adds defects that serve as recombination centers. Nano cobalt oxide was found in comparative analysis with nanostructured metal oxides (cobalt, titania and iron) to be significantly faster in hydrogen generation rate compared to nano titania oxide Moreover, nano ferrite had a rate which was almost four times more than that of nano cobalt oxide (Mangrulkar et al., 2012).

With a broad direct bandgap of 3.37 eV and a high exciton binding energy, zinc oxide (ZnO) is an n-type semiconductor that may be used in a variety of optoelectronic applications. Its broad bandgap, however, drastically limits its efficacy in water splitting by confining light absorption to the UV spectrum, which makes up just 4% of solar energy. Inadequate surface reactivity and the quick recombination of photo-generated carriers also cause ZnO to perform poorly. These inherent disadvantages emphasize the need to use low-dimensional nanostructures, which provide greater surface areas and shorter migration routes to improve catalytic efficiency. Therefore, even though undoped ZnO has advantageous physical characteristics, its optical and kinetic restrictions prevent it from being used in solar energy conversion (Ma et al., 2020).

Similar to TiO_2_, ZnO is a top photocatalyst with benefits such a similar bandgap, better electron mobility, and fewer defects. It also has a special piezotronic effect, in which mechanical strain creates an internal electric field that facilitates charge separation. Systematic investigations demonstrate that doping can create high-quality p-type ZnO, even if it is still difficult to form robust p-n junctions for commercial usage. By adding additional band energy levels, this doping not only increases light absorption into the visible spectrum but also makes it possible for homojunctions and heterojunctions to form. By using inherent electric fields to speed up charge separation, these junctions greatly increase the effectiveness of photocatalytic water splitting (Table 2; Cao et al., 2022).

**TABLE 2:** Summary of the optical properties, photocatalytic performance, and key challenges of undoped semiconductor photocatalysts used in water splitting.

Material	Bandgap (eV)	Light absorption	Advantages	Key challenges	References
Sc_2_O_3_	∼5.6–6.0	Deep UV only	Extremely high chemical and thermal stability; non-toxic	Cannot function as a photoactive semiconductor host	Zhang et al. (2019)
TiO_2_	3.05–3.2	UV only	High stability, non-toxic, excellent charge transport	Cannot absorb visible light (∼4–5% of spectrum)	Ni et al. (2007)
MnO_x_	Variable (e.g., Mn_2_O_3_ ∼2.2 eV)	Visible light	Excellent visible light harvesting; earth-abundant; highly active as a co-catalyst for water oxidation	Poor conductivity and severe dissolution (corrosion) in aqueous electrolytes	Robinson et al. (2013)
Cr_2_O_3_	∼3.0–3.4	UV to near-UV.	Resistance to photocorrosion (structurally)	Limited UV absorption; very poor surface reaction kinetics. Also, risk of oxidation to toxic Cr(VI) under harsh potentials	Kasikov et al. (2019)
ZnO	3.37	UV only	High electron mobility, piezotronic effect	Photo corrosion, fast recombination	Liu et al. (2017)
α-Fe_2_O_3_ (hematite)	∼2.0	Visible light	Abundant, stable	Poor optoelectronics, short hole diffusion	Tokubuchi et al. (2021)
V_2_O_5_	Small (visible)	Visible light	Suitable conduction band for H_2_ evolution	Fast recombination of carriers	Li et al. (2023)
Cu_2_O	∼2.0–2.2	Visible light	Low cost, suitable band edges	Photo-corrosion (instability)	Tilley (2023)

One important instability mechanism in ZnO is photo corrosion, which is defined by the material’s self-decomposition via hole-induced oxidation. By lowering the concentration of positively charged holes, this deterioration can be lessened. The improved photo corrosion resistance in this work is explained by the ZnO lattice’s decreased surface defect sites, which in turn reduces the availability of holes that accelerate the deterioration process (Zhang et al., 2014). When exposed to radiation, photo-generated electrons from the carbon matrix go to nickel centers and surface defects to promote proton reduction. Because nickel has a larger work function than carbon, it functions as a better catalyst. Concurrently, the incorporation of a lactic acid sacrificial donor increases the reduction of Ni^2^_+_ to Ni^0^ and enhances charge separation, resulting in the creation of additional active sites that greatly increase the overall catalytic efficiency (Mijowska et al., 2024).

### 2.2 Effects of transition metal oxide as dopants in photocatalytic splitting of water

Transition Metal Oxide (TMO) dopants cause their control on photocatalytic water splitting by making intrinsic changes to the crystal structure of the semiconductor and electronic band structure. Addition of transition metal cations (e.g., Fe^3+^, Mn^2+^, Co^2+^, Cr^3+^, V^4+^) into the host lattice (e.g., Ti_2_O_3_ itself or SrTiO_3_) has various effects on the material regarding the response to light and charge transport.

#### 2.2.1 Sacandium

By adding shallow acceptor levels between 10 and 25 meV above the Fermi level, scandium doping causes p-type behavior in TiO_2_. This doping causes holes to localize while also increasing the density of states in the valence band. The spontaneous dissociation of electron-hole pairs is driven by this hole localization, which significantly increases the efficiency of solar cell systems (Singh et al., 2023). A localized internal electric field is created inside the rutile TiO_2_ lattice via Sc doping. As soon as bound electron-hole pairs (excitons) are created, this field causes them to spontaneously dissociate into free charge carriers. The arrangement keeps the density of active charge carriers high by stopping the fast recombination that is characteristic of undoped TiO_2_. An impressive apparent quantum yield of 30% for total water splitting is made possible by this effective charge separation process, which is a significant improvement over undoped rutile (Qin et al., 2025).

#### 2.2.2 Vanadium

When nano-rutile TiO_2_ doped with V^5^_+_, lattice contraction is induced and visible light absorption is much increased, improving catalytic stability. Excessive doping lowers photocatalytic activity, although oxygen evolution rises with dopant concentration up to an ideal limit. By generating donor levels close to the conduction band (CB), V^5^_+_ promotes electron transport and increases the lifetime of vacancies. In the end, this structural and electrical change increases the water-splitting reaction’s efficiency and promotes oxygen evolution rate (OER) (Wu et al., 2011).

The effect of the vanadium doping concentration on the photocatalytic performance is concentration-dependent. When incorporated at low to moderate levels, V will create defect levels or donor states near the conduction band (CB) which will increase the light absorption range towards the visible spectrum and enhance the efficiency of the photoexcitation process. However, these dopant states may be recombination active under high vanadium loadings particularly when the dopant forms a secondary phase, causes oxygen vacancy, or causes lattice distortion. The effect of V doping is not necessarily beneficial or detrimental, but depends on an optimum combination of improved absorption and reduced recombination (Liu et al., 2009).

The comparative study of V-doped TiO_2_ nanoparticles and thin films, performed based on the femtosecond transient absorption spectroscopy and photo electrochemical analyses, demonstrates the similarity of the mechanistic findings on the influence of Vanadium. V doping has been effective in both morphologies in introducing a localized intra-gap state at about 2.2 eV below the conduction band (CB) minimum. The level of impurity allows sub-bandgap photoexcitation at near 530 nm, in effect extending the photocatalytic activity into a spectral range of the visible spectral region not actively photo catalyzed by undoped TiO_2_. Nevertheless, these two studies come to a common agreement on a very important trade-off; the introduction of this state will strongly speed up the kinetics of electron-hole recombination. As a result, although V-doping enables the use of visible light, the increased rates of recombination cause an observable reduction in conversion efficiency to the energy, especially in the UV spectrum (Piccioni et al., 2020; Rossi et al., 2018).

Particularly when co-doped with nitrogen, V^4+^ substitution in TiO_2_ produces isolated impurity levels 0.8–1.0 eV below the conduction band, which further pushes light absorption into the visible range. Furthermore, photo-generated electrons from TiO_2_ are instantly transferred to the V^5+^ species due to the formation of a space charge layer at the interface caused by the presence of V^5+^ ions. Rapid charge transfer is made possible by this process, but excessive V_2_O_5_ buildup, which is seen at higher V/Ti ratios, can block active sites and reduce photocatalytic performance (Jaiswal et al., 2012). Vanadium doping produces oxygen vacancies that can operate as recombination sites and V 3 days states below the conduction band that function as electron traps, encouraging charge separation. Codoping with nitrogen provides a synergistic solution, even though vanadium is very successful in reducing the band gap to improve visible light absorption. By filling the oxygen vacancies that vanadium creates in V-N co-doped systems, nitrogen lowers recombination rates and greatly increases the lifespan of charge carriers (Varma et al., 2018).

#### 2.2.3 Chromium

Chromium doping in SrTi-Cr_x_O_3_ produces two different visible-light absorption features: a wide band (540–800 nm) resulting from d–d transitions inside octahedral Cr^3^_+_ centers and a peak at 550 nm attributable to charge transfer from Cr^3^_+_ to Ti.^4^_+_ These characteristics allow charge transfer upon activation and are made possible by a hybrid Cr 3 d–Ti 3ays electronic structure. Because chromium increases the range of light absorption across various impurity states, the measured photocatalytic performance is ultimately directly associated with the quantity of chromium (Liu et al., 2006).

With ZnO-based photocatalysts, Chromium (Cr) is a concentration-dependent ion with a dual nature (that is, it can be either beneficial as an electronic modifier (as Cr^3^_+_) as a recombination center as Cr^6^_+_. The loss of Cr^6^_+_ TOCr^3^_+_.causes the appearance of donor states in the bandgap, which increases the visible light absorption and charge separation, thus, improving photocurrent density. On the other hand, the existence of Cr^6^_+_ also serves as a recombination site that deteriorate performance. This results in positive correlation between Cr concentration (to 15 percent) and efficiency, with 15 percent Cr-doped ZnO showing the highest photocurrent density (866 -mu A/cm^2^) and the best stability because it can effectively prevent photo electrode corrosion (Khan et al., 2020).

Uniformly loaded Mn_3_O_4_ nanoparticles on both SrTi_3_ and BiVO serve as the effective Oxygen Evolution Reaction (OER) centers, and the activity of these centers also promoted by the addition of Cobalt. In particular, the flat band potential not altered significantly by low concentrations of Co-doping (it is no less than 40 mol). The subsequent increased photocatalytic performance is, therefore, due to the development of active clusters of the active material, i.e., Co_x_Mn_3-x_O_4_ on the surface, which do not enhance charge separation but instead maximizes surface reaction kinetics (Yoshinaga et al., 2018). Mn-doped TiO_2_ films show a drop in bandgap energy proportionate to the manganese content and a red shift in the absorption edge when compared to naked TiO_2._ This bandgap narrowing increases the material’s capacity to produce hydrogen during solar water splitting by extending light absorption into the visible spectrum (Momeni et al., 2016).

#### 2.2.4 Cobalt (Co)

Co was found by Jang et al. to be the most effective dopant to OER amongst Mn, Fe, and Ni. It has the lowest Tafel slope and over potential. The stabilization of the active Co^3+^ ions is provided by the oxide matrix which allows adsorption of OH-ions and O-O bond formation (Jang et al., 2015). When a cobalt oxide film integrated with alpha- Fe_2_O_3_ the power needed to drive the catalytic process significantly reduced. By using photo-generated, holes in an n-type semiconductor, like ZnO, to oxidize Co^2+^ to Co^3^_+_ cobalt oxide deposition may also be accomplished photo chemically. The cobalt oxide catalyst carefully positioned at the surface where holes are created using this photo deposition process, especially at the interface where oxygen evolution is most efficient. Consequently, the water photo-oxidation over potential was reduced by 0.2 V and the photocurrent was somewhat increased in this novel composite photo anode (Artero et al., 2011). By serving as an excellent hole-trapper for better charge separation and transportation, a larger ratio of Co^2+^ in the amorphous CoO_x_ co-catalyst considerably increases the photocurrent adaptability. Theoretically, the electrical structure of the Co^2+^ species (3 d^7^) makes it more vulnerable to electron loss and hole abstraction than the Co^3+^ species (3 d^6^). Greater Co^2+^ ratio, exhibits better efficiency as an OER photocatalyst (Xiao et al., 2021).

The results of quantum-chemical calculations and previous reports suggest that the oxygen-vacancy formation energy significantly reduced upon Fe substitution in comparison with Mn and Co substitution. This thermodynamic preference drives the formation of Fe vacancies complexes, which produce a higher local lattice distortion, modify the electronic density around the Fermi level and encourage a defect mediated charge carrier response. However, in the case of substitutions of atoms such as Mn and Co., the host lattice is retained largely, which leads to more retention of the host lattice from the substitutions, thus the contribution is mainly through moderate band-structure modulation and less through vacancy stabilization. Therefore, the different experimental trends in Fe-, Mn- and Co-doped systems can be understood based on the different defect chemistries and electron structures of the systems.

#### 2.2.5 Nickel

Stronger interfacial bonds cause single Ni atoms to preferentially adsorb onto the TiO_2_ surface, while multiple Ni atoms often accumulate on the SrO surface before scattering onto the TiO_2_ surface. Ni cluster deposition strengthens the semiconductor’s n-type character and promotes the Hydrogen Evolution Reaction (HER) by shifting the Fermi level toward the conduction band. The (NiO) _n_ clusters are structurally similar to the SrTiO_3_ surface, and their derived states overlap the valence band. This alignment permits holes in the valence band to migrate to the clusters under light irradiation, which facilitates electron-hole pair separation and improves photocatalytic performance (Wang et al., 2019). In comparison to the incipient wetness impregnation approach, single-step sol-gel synthesis produces homogeneous and well-dispersed NiO on the TiO_2_ substrate, which leads to noticeably enhanced photocatalytic hydrogen evolution activity. The effective movement of photo excited electrons from the TiO_2_ conduction band to NiO trapping sites is responsible for this improved performance. Two key components contribute to this system’s superiority: the high dispersion of the NiO cocatalyst maximizes the interfacial contact between the cocatalyst and the support, and the mesoporous network of the TiO_2_ support enhances reactant mobility (Ali and Najmy, 2013).

Compared to pure TiO_2_, coupling TiO_2_ with modest NiO loadings (0.1% and 0.5%) greatly increased the photocatalytic reduction of Cr (VI). The creation of a p-n junction, where the inner electric field effectively separates electron-hole couples by pushing holes to the NiO phase and delaying recombination, is responsible for this increase. Nevertheless, photocatalytic activity was reduced when the NiO dosage was raised over 1% because excessive NiO loading functions as a recombination center that prevents charge transfer and causes electron-hole recombination (Ku et al., 2011).

Strong O-Ni hybridization in the (NiO) _n_ clusters shown by electronic structure analysis using HSE06 TDOS and PDOS, which is suggestive of covalent bonding. These cluster states exist at a higher energy level even when they cross the host valence band (VB). As a result, electrons move from the cluster to the host valence band (VB) upon photoexcitation of the host semiconductor (Ga_2_O_3_). The Oxygen Evolution Reaction (OER) can be fueled by the holes left in the clusters by this migration. Nevertheless, a large energy barrier is created by vacant Ni levels located 4.5 eV above the Fermi level, making the Hydrogen Evolution Reaction (HER) thermodynamically unfavorable (Liu T. et al., 2015). Consistently, the Co/NiO_2_ exhibits significantly reduced semicircle radii and higher photocurrent intensity, making it the best at driving charge transfer Co/NiO_2_ rich deficient structures and single-crystal structure are likely responsible for the effective separation and transfer of charge carriers. The deficient structure can act as active sites to prevent electron-hole recombination by trapping electrons from Ru, while the single-crystal nature enhances charge transfer (Zhang et al., 2023).

#### 2.2.6 Zinc

When divalent cations (especially Zn^2+^) were added to Ga_2_O_3_, the activity was significantly increased by almost 20 times when compared to Ni/Ga_2_O_3_. This enhancement was attributed to the addition of a Zn site, which increased the mobility and concentration of photo-generated holes caused by an electronic reconstruction (Che Mohamad et al., 2021). The importance of Zn-doping and interface engineering in improving transition metal oxides for effective water splitting highlighted by this work (Table 3). Zn doping allows for exact customization of the effective masses of charge carriers by positively altering the electronic structure of the valence band (VB) and conduction band (CB). The decrease in electron effective mass and the notable difference in the effective masses of electrons and holes are responsible for the better water-splitting performance shown in the 9% Zn-doped NiO sample created under vacuum (Kiran et al., 2023). TiO_2_–ZnO mixed oxides have been created utilizing the sol–gel technique in order to increase Titania photocatalytic efficiency for water splitting. Because of its advantageous valence and conduction band locations, which are thermodynamically appropriate for water redox processes, zinc oxide chosen as a co-catalyst. Additionally, by lowering the rate at which photo -generated electron-hole pairs recombine, the addition of ZnO is crucial in limiting charge recombination (Pérez-Larios et al., 2012).

**TABLE 3:** Overview of transition metal oxide (TMO) dopants, their host materials, primary charge-transfer mechanisms, and impact on photocatalytic performance.

Dopant	Host material	Primary mechanism	Effect on performance	Wavelength range	Sacrifical agent	Key observation	References
Scandium (Sc)	TiO_2_ (Rutile)	Type I/junction-assisted charge separation	High charge separation	Xe-lamp, 360 nm	None(pure water)	Achieved 30% apparent quantum yield (AQY)	Qin et al. (2025)
Vanadium (V)	TiO_2_	Not a true heterojunction; defect-mediated band engineering)	Extends absorption to ∼530 nm	Vis light, >450 nm	Not specified	Increases visible light but accelerates recombination	Rossi et al. (2018)
Chromium (Cr)	SrTiO_3_/ZnO	Defect/band-state engineering	Enhanced visible absorption	AM 1.5 simulator	None (PEC/bias)	Concentration dependent; Cr^3+^ is beneficial, Cr^6+^ is detrimental	Qin et al. (2024)
Manganese (Mn)	TiO_2_/Mn_3_O_4_	Type II or composite-assisted charge transfer	Red shift in absorption	Xe-lamp (UV)	None (pure water)	Co-doping with Co. further boosts OER activity	Momeni et al. (2016)
Cobalt (Co.)	α-Fe_2_O_3_/ZnO	Type II or S-scheme depending on band alignment	Lowers OER overpotential	Xe-lamp, ≥420 nm	AgNO_3_ (Ag^+^)	Co^2+^ species show better hole-capturing than Co^3+^	Xiao et al. (2021)
Nickel (Ni)	TiO_2_/SrTiO_3_	p Type II/p-n junction-assisted transfer	Improves H_2_ evolution	UV light	Ethanol	Optimal at low loading (0.1%–0.5%); high loading causes recombination	Ku et al. (2011)
Zinc (Zn)	NiO/Ga_2_O_3_	Interface engineering, not necessarily Type I/II	Enhances hole mobility	NR	NR	Doping modifies effective mass of charge carriers	Kiran et al. (2023)
Copper (Cu)	TiO_2_ Nanotubes	Type II heterojunction/p-n junction	20%–50% increase in photocurrent	AM 1.5G simulator	None (PEC)	Forms efficient junctions for charge separation	Wang et al. (2020)

#### 2.2.7 Copper

Water splitting in PEC cells greatly improved by decorating Titanate/TiO_2_ nanotubes (TNTs) with CuO nanoparticles. These results in performance gains of 20% under AM 1.5G filtered light and 50% under an open spectrum. The main cause of this enhancement is the temporary creation of a p-n junction between the TNT structure and the Cu_x_O nanoparticles under light, which promotes effective charge separation (de Brito et al., 2018). Introduction of Copper Oxides (Cu and Cu_2_O) into TiO_2_ nanotubes forms a highly efficient Type II heterojunction system of photocatalyst of water splitting which essentially separate the charges and inhibits recombination (Nikhila et al., 2023).

Copper really plays a multifaceted function in this system: it creates a large concentration of advantageous defects and oxygen vacancies that enhance carrier separation, and it greatly alters the TiO_2_ lattice, as seen by the Raman shift. It efficiently increases visible light absorption (400–800 nm) and narrows the bandgap, extending light absorption up to 800 nm. Most importantly, copper effectively traps photo-generated electrons, delaying the rate of recombination and thereby increasing carrier lifetime (Yan et al., 2020). Cu_2_O is a cheap, non-toxic semiconductor that can be used for visible light-driven water splitting because of its small 2.17 eV bandgap. When combined with CuO, the combination exhibits band edges that are in good alignment with both hydrogen and oxygen evolution activities and extends absorption into the near-infrared spectrum. However, a significant drawback is that the material is vulnerable to photo degradation through simultaneous oxidation and reduction interactions since its redox potentials fall inside the bandgap (Baran et al., 2021). CuO quantum dots greatly increase photocatalytic performance mainly through improved charge transfer by facilitating the separation of photo-generated charges and preventing electron-hole recombination. Furthermore, the unaltered Cu^2+^ XPS peak, which shows that the CuO placed on TiO_2_ stays stable with minimal photo corrosion, confirms the stability of the composite (Wang et al., 2020).

### 2.3 Light absorption and bandgap engineering

TMO doping generates a variation of optical bandgap (Eg) as the strongest effect. This is because the presence of partially filled d-orbitals of transition metals creates intermediate levels (impurity bands) in the forbidden gap of the host semiconductor. This practically decreases energy to excite the electrons to the conduction band (CB) through the valence band (VB).

#### 2.3.1 Chromium (Cr)

Dholam et al. have shown that Cr-doping is quite helpful in bandgap narrowing, to decrease bandgap of. TiO_2_ even up to 2.1 eV at 100 percent concentration (9at%) (Dholam et al., 2010). Doping TiO2 with Cr^3+^ enables visible-light oxygen evolution, where Cr^3+^ narrows the bandgap while Sb5+ co-doping prevents lattice defects by inhibiting Cr^6+^ formation. However, this ion implantation technique is limited by high cost and low quantum yields (Mohsin et al., 2023). This enables the material to absorb the visible light like to a depth of about 600 nm which has a tremendous extension on the solar spectrum usage than the pure material (=3.2 eV).

#### 2.3.2 Manganese (Mn) and iron (Fe)

According to Mahmoud et al. Mn doping decreased the bandgap of titanate nanotubes to 2.81 eV, which improved the ability of titanate nanotubes to sense light by 35 percent in the visible range (Mahmoud et al., 2018). Equally, Wang et al. have found that Fe-doping reduced the bandgap of. TiO_2_ nanorods to 2.15 eV (Wang et al., 2014).

#### 2.3.3 Vanadium (V)

Bantawal et al. demonstrated that V-doping of SrTiO_3_ decreases the bandgap to be 2.54 eV, which spans into the visible range, whereas still preserving the perovskite structure (Bantawal et al., 2020).

#### 2.3.4 Copper

Cu doping resulted in a 21.9% increase in visible light absorption and a band gap reduction to 3.2 eV. It creates a p-type CuO phase on the nanotubes surface. As a result, the n-type titanate and p-type CuO form a coupling that facilitates charge separation by encouraging the transport of electrons and holes to the interface (Mahmoud et al., 2018).

### 2.4 Dynamic of charge carrier and recombination

TMOs enhance light harvesting, but their role on the charge carrier dynamics is difficult and composite in nature. Impurity levels brought in by d-orbitals may be either shallow traps (beneficial) or deep recombination centers (detrimental).

#### 2.4.1 The Mn and Co phenomenon (trap)

It was observed that Mn and Co doping were able to reduce the bandgap gap, but reduced the density of charge carriers as well. Mott-Schottky analysis revealed that Mn and Co provided defect states that were the recombination centers and prevented the movement of electrons to the surface. This implies that in the specific metals, the increase in the absorption of light offset by the decrease in mobility of charge. Consistently, the Co/NiO_2_ exhibits significantly reduced semicircle radii and higher photocurrent intensity, making it the best at driving charge transfer. Co/NiO_2_’s rich deficient structures and single-crystal structure are likely responsible for the effective separation and transfer of charge carriers. The deficient structure can act as active sites to prevent electron-hole recombination by trapping electrons from Ru, while the single-crystal nature enhances charge transfer (Zhang et al., 2023). Fe-doping was discovered to be more effective (unlike Mn and Co) in raising the density of carriers. The ability of Fe-doped samples to attain photocurrent densities that are fivefold that of undoped samples allows Fe to be a better dopant than any first-row transition metal (Wang et al., 2014).

The effect of the dopants can be very different because of the type, oxidation state, concentration, lattice position and synthesis route of the dopants. Dopants can in some cases form shallow electronic states that facilitate the absorption of visible light and charge separation, and in other cases, they can form deep trap states that lead to recombination and decrease activity. The variability of the performance results between different studies is frequently associated with differences in the location, dispersion, and concentration of the defects, in the ratio of secondary phases, and not just with the type of dopant. Thus, the overall conclusion of these studies is that doping should not be general but well-optimized, in order to consider the balance between the effect of the band-gap modification, the carrier lifetime, and structural stability.

### 2.5 Effect of carbon based materials

Carbon-based nanostructures are very successful in a variety of photo-based applications, such as organic catalysis, water splitting, hydrogen generation, and CO_2_ reduction. Attractive qualities including cost effectiveness, environmental friendliness, and superior chemical and thermal stability are what drive their value. They are also perfect materials for these cutting-edge technologies because to their low framework density and ease of processing (Khan, 2021). The use of carbonaceous material to improve semiconductor photo catalysis by inhibiting recombination of charges and providing a hydrophobic microenvironment that concentrates the reactants around active sites is on the increase. Recent report also suggests that graphitic carbon can be used as a sensitizer if it forms an efficient interface with TiO_2_. This is a two-way functionality, which enhances charge dynamics and light harvesting of photocatalyst composites (Ng et al., 2012). Synergistic interactions between photocatalysts and carbon materials through high surface area to enhance dispersion and broaden of electrons to transit through the system to the product enhance yield of the product with the carbon materials. Although GCN and graphene possess different electrical properties, for example, GCN is a semiconductor and graphene is a conductor, the two materials are good conducting supports. This combination can be used to increase the efficiency of photocatalytic CO_2_ removal significantly (Kandy, 2020).

#### 2.5.1 Graphene

The reduced graphene oxide (RGO) is one of the cost-effective, high-conductivity mediums with large surface area that supports the excellent electron transfer. RGO facilitates charge carrier transfer by creating heterojunctions with the photocatalysts or facilitates charge carrier transfer with the photocatalysts largely, minimizing recombination rates, which results in a high photocatalytic efficiency (Tahir et al., 2021). Because of its large surface area and defect sites, which serve as metal oxide nanoparticle nucleation sites, graphene is a great supporting material for semiconducting photocatalysts. In particular, these nanoparticles are drawn to and immobilized by the negatively charged surface of reduced graphene oxide (rGO), which stops them from aggregating. This structural stability greatly increases the photocatalyst’s longevity and reusability (Fadlalla and Babu, 2019).

**FIGURE 5:**
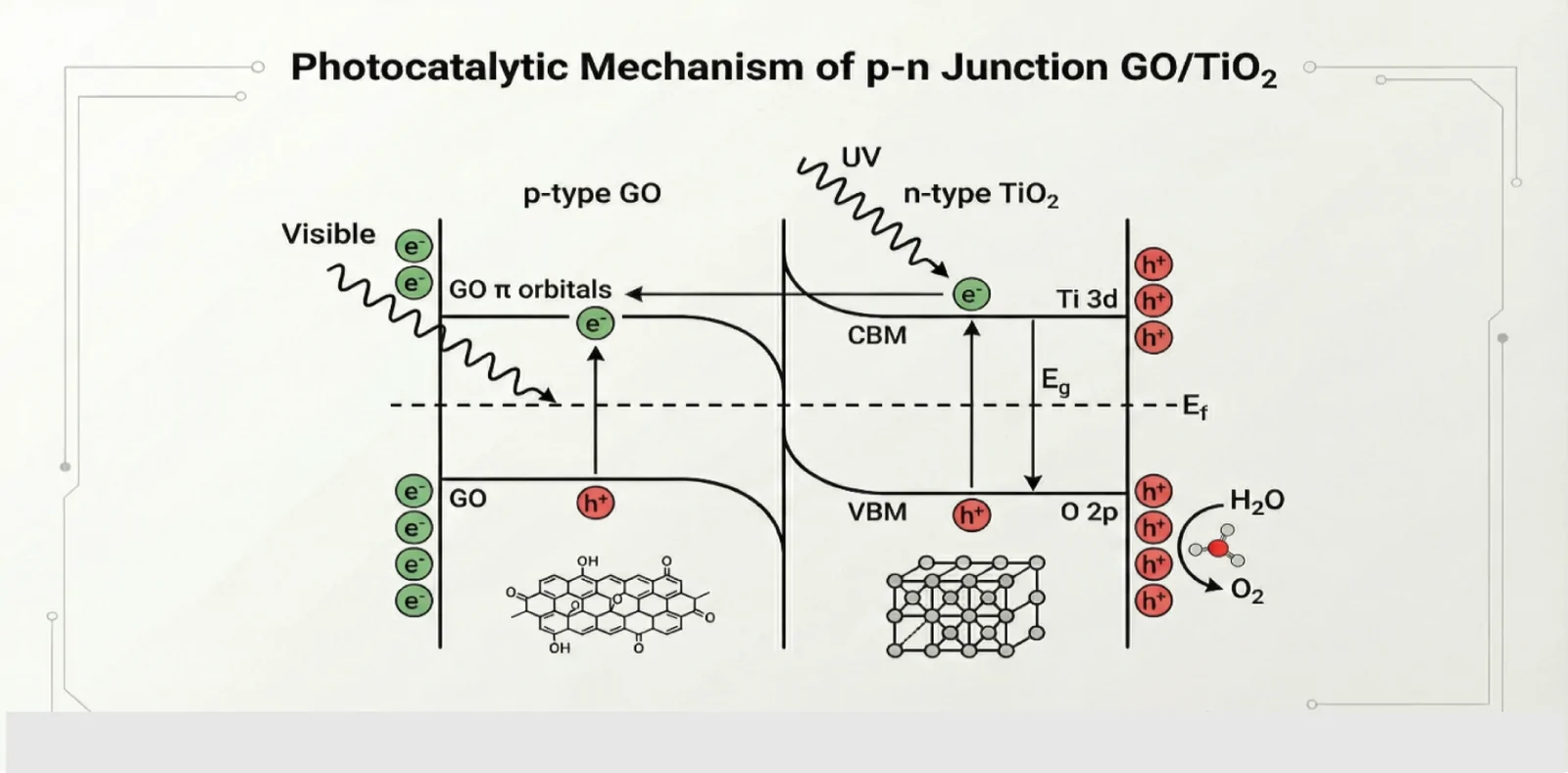
Schematic illustration of the mechanism of the p-n junction of GO/TiO_2_. This figure was generated by the authors using Genspark and NotebookLM, and subsequently edited and annotated using Adobe Illustrator.

Although the zero band gap in ideal graphene results in quick recombination of charges, oxidation transforms it to GO or r-GO that are semiconductors with adjustable bandgaps (2.443 eV). GO sheets are versatile 2D soft matter that improve photo catalysis in a number of ways. In addition to establishing p-n junctions to further improve charge separation, GO-based materials a used as sensitizers (Figure 5). Moreover, GO functions as an electron transport mediator to support the charge separation in the system such as Z-scheme photocatalysts. Moreover, p-electrons on GO that are not paired with other atoms may react with wide-bandgap semiconductors such as TiO_2_ to expand their light absorption. Or, the p-type nature of GO permits the creation of p-n junctions with n-type semiconductors, in which the internal electric field causes electrons to be injected into the semiconductor as holes are transferred to the semiconductor by the GO (Albero et al., 2019; Yeh et al., 2013). When graphene is present, charge separation efficiency increases (Krishna et al., 2023).

The presence of CuO and graphene together with TiO_2_ results in a synergistic action that improves the quantity of hydrogen generated through photo catalysis by reducing the recombination of charges. Graphene serves as an acceptor of electrons and conduction highway, where photo-generated electrons of TiO_2_ to CuO active sites are transferred. These electrons combine with the protons on the CuO surface to form the hydrogen and the holes oxidize methanol. The mechanism of this multi-pathway charge transfer is very efficient in the separation of electrons and holes (Ragupathi et al., 2020). In addition to having a low rate of electron-hole pair recombination and a quick transfer of photo induced carriers, graphene shows outstanding performance in reducing photo corrosion of Cu_2_O (Dubale et al., 2014).

**FIGURE 6:**
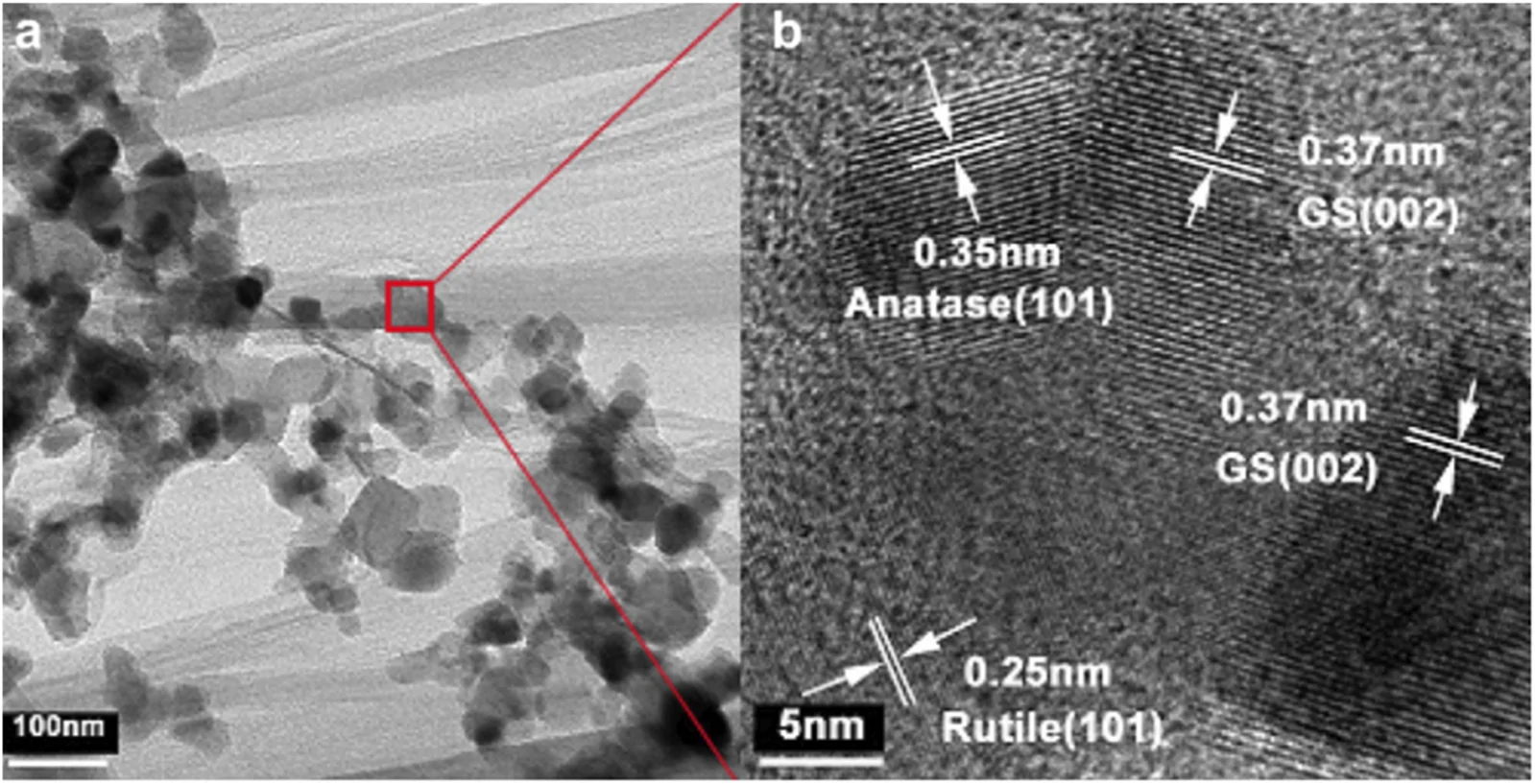
TEM image **(a)** and HRTEM image **(b)** of P25e10%GR (Cheng et al., 2012).

Graphene enhances photocatalytic water splitting by acting as a conductive electron sink and p-n junction mediator that suppresses recombination, extends visible light absorption, while its high-surface-area support prevents agglomeration, and ensures efficient charge transport to active sites. TEM and HRTEM images reveal the dispersion of 20–30 nm P25 nanoparticles on wrinkled sheets of graphene and lattice fringes of anatase, rutile, and graphene phases (Figure 6). Patterns of XRD confirm the existence of anatase and rutile TiO_2_ crystal structure in the composites. The contact between the P25 and graphene reduces the band gap and increases light absorption with increase in the content of graphene. These structural and optical enhancements, therefore, result into an increase in photocatalytic hydrogen evolution activity as opposed to bare P25 (Cheng et al., 2012).

#### 2.5.2 Carbon nanotubes

Carbon nanotubes (CNTs) are good choices as photocatalytic composites, and their hollow cores and outer walls give them an extremely high surface area. Their superior electrical conductivity serves as a sink of electrons and thus transfers photo carriers to the semiconductor surface rapidly, and the charge separation significantly enhanced. In addition, CNTs exhibit high Van der Waals bonding to adsorb reactants because of their tubular structure, and a network to transfer charges rapidly between the electrolyte and semiconductor (Alenad et al., 2023). Carbon nanotubes (CNTs) may find use in Z-scheme systems as mediators. These materials can facilitate effective charge transfer since they are very stable and have good conductivity (Lin L. et al., 2024).

The addition of CNTs (CNTs@TNRs) causes a notable redshift into the visible light spectrum, whereas pure TNRs only show absorption in the UV range. Because of the CNTs’ large surface area and strong conductivity, this electrical structural alteration lowers the band gap from 3.78 eV to 2.07 eV and improves electron transfer efficiency (Ahmed et al., 2020). Incorporating CNTs into graphene-supported TiO_2_ creates a hierarchical nano hybrid where CNTs enhance electron transport, prevent graphene restacking, and increase surface area. This structure drastically boosts photocatalytic hydrogen production (8-fold vs. P25) by minimizing recombination through Ti^3+^ sites, oxygen vacancies, and strong interfacial contacts that narrow the band gap. The resulting CNT-GR-TiO_2_ composite facilitates efficient electron flow from TiO_2_ through the carbon network, proving to be a stable, reusable, and highly active catalyst (Bellamkonda et al., 2019).

**FIGURE 7:**
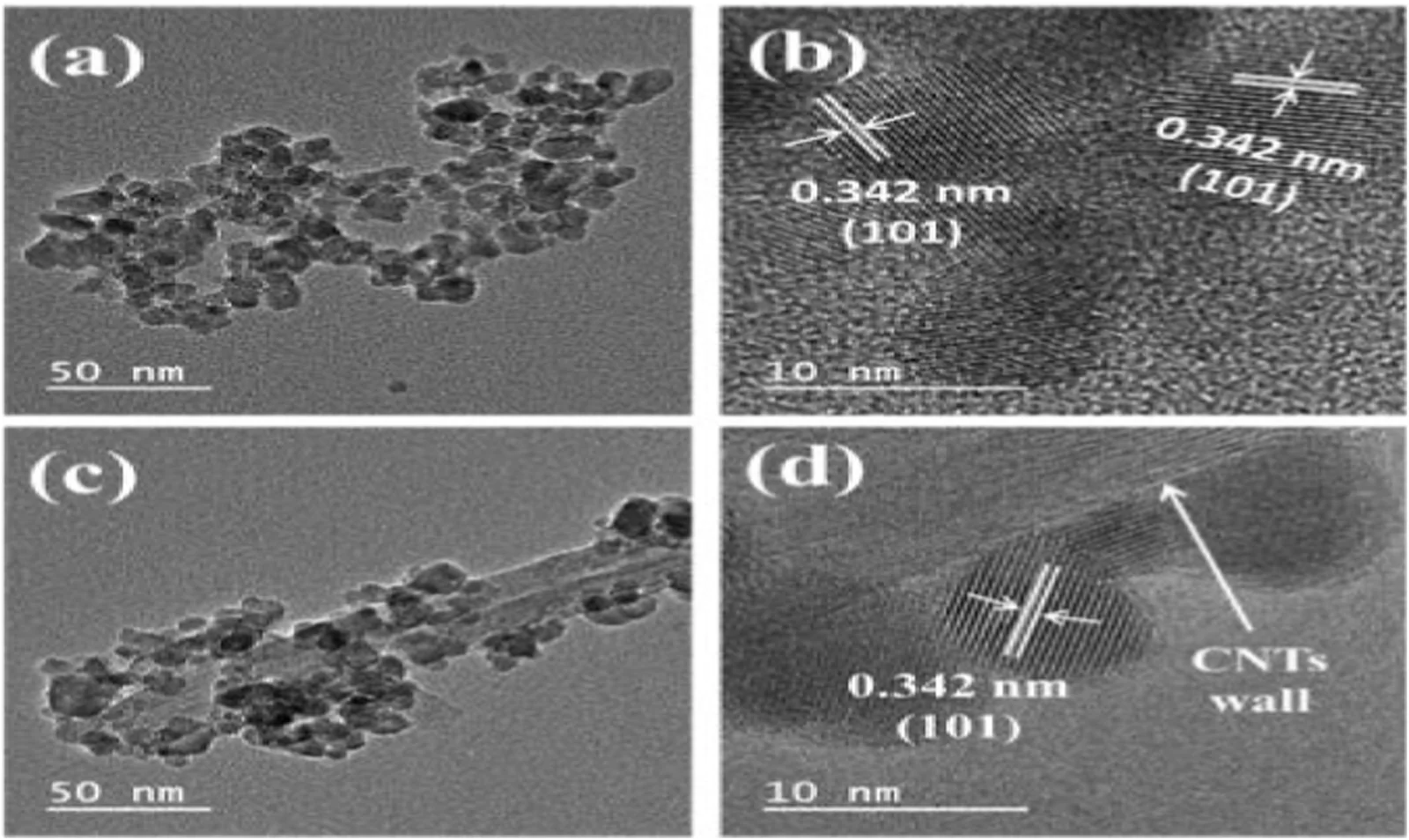
TEM and HRTEM images of the synthesized **(a,b)** TiO_2_ and **(c,d)** 2.0CNT−TiO_2_ (Olowoyo et al., 2018).

Because of its cheap cost and non-toxicity, nanostructured ZnO is a potential material for both PEC water splitting and photocatalysis; nevertheless, fast charge recombination limits its efficiency. This is solved by combining ZnO with carbon nanotubes (CNTs) to create nanocomposites in which CNTs serve as effective electron acceptors and offer a wide surface area and distinctive one-dimensional structure. Therefore, it is expected that ZnO/CNT nanocomposites would perform much better in photocatalytic processes and PEC water splitting (Chaudhary et al., 2018). The microstructure of pure and TiO_2_/CNT has been analyzed by TEM. The findings revealed that pure TiO_2_ is composed of spherical nanoparticles the average size of which is about 12 nm (Figure 7). When CNTs were added, the size of the particles was reduced to 7nm, which confirms that CNTs suppress the growth of TiO_2_ particles-a result similar to that of XRD data. The images showed the presence of spherical TiO_2_ particles attached to the walls of the CNTs, which forms a tree-shaped structure at a higher concentration of the CNTs. This morphology suggests that it has a high degree of physical and chemical interactions that improve electron and ion transportation at the interface. Also, the presence of an anatase form of TiO_2_ was verified by the HRTEM analysis that revealed the lattice fringe spacing of 0.342 nm, which is the (101) plane (Olowoyo et al., 2018).

#### 2.5.3 Activated carbon

Activated carbons increase the photocatalytic process due to its physical and chemical characteristics, especially, large specific surface area. This aspect enhances contact area between the catalyst and pollutants hence becoming more efficient through the carbon being able to serve as an effective porous support (Velo-Gala et al., 2013). Activated carbon (AC) has a surface which consists of 3D fused aromatic rings and this allows it to chemisorbed by transfer of electrons instead of physiosorbed. AC has high absorption of light and is hence suggested to act as a solid photosensitizer, thus increasing the overall photoactivity of the system (Murcia-López et al., 2013). Carbon-doped TiO_2_ has a much greater photocatalytic activity compared to nitrogen-doped or undoped counterparts, and up to thirteen times greater activity by being able to absorb visible light. This increase in performance can be blamed on large bandgap narrowing - to as small as 1.45 eV - and the creation of mid-gap states that push photo response all the way to the near-infrared band.

Theoretical and experimental literature attest to the fact that carbon doping does not only modify the electronic structure, but it also enables effective water splitting and general photoactivity (Table 4; Shaban and Khan, 2008). The general findings are that the activated carbon (AC) is an inherent semiconductor and solid photosensitizer, and can produce electron-hole pairs on irradiation by virtue of a fused aromatic ring structure. The holes and electrons are not a passive adsorbent but take an active part in redox reactions to form hydroxyl and superoxide radicals. It emphasizes the fact that surface chemistry of AC is of extreme importance because presence of oxygen-bearing functional groups on this surface is a trap of electrons that creates charge separation and encourages chemisorption in favor of physisorption. Therefore, the AC makes good use of its conductive carbon matrix and surface sites of active sites to promote photocatalytic oxidation activities under UV and solar light (Velo-Gala et al., 2017).

**TABLE 4:** Quantitative photocatalytic and photoelectrochemical performance metrics of transition-metal- and carbon-based dopant/modifier strategies for water splitting.

Dopant/Modifiers	Host material	Light source	H_2_ evolution rate (µmol h^-1^ g^-1^)	Photocurrent density (mA/cm^2^)	Apparent quantum yield	Solar-to-hydrogen conversion efficiency	References
Scandium (Sc)	TiO_2_ (Rutile)	Xe lamp 360 nm	NR	NR	30%	0.34	Qin et al. (2025)
Chromium (Cr)	SrTiO_3_/ZnO	400 nm	24.4 μmol h^-1^	41.6	1.61% at 400 nm	0.03%–0.4%	Dholam et al. (2010)
Manganese (Mn)	TiO_2_//ZnO	Visible light region	1,376–1778	0.84	1.6	0.3–0.5	Das et al. (2023), Gurudayal et al. (2014), Pérez-Larios et al. (2016)
Cobalt (Co)	TiO_2_/SrTiO_3_	420 nm	220	3.45	27.6% at 420 nm	1.57%	Mishra et al. (2023), Sadanandam et al. (2013)
Iron (Fe)	TiO_2_/CuO/ZnO	420 nm /xenon lamp	15.5 μmol/h	1.3	155 times grater than CoO	5.98%	Dholam et al. (2009), Rahmawati et al. (2024), Shaban et al. (2024)
Nickel (Ni)	TiO_2_	UV light	180	4.5	0.5% to over 15%	2.95%	Ismael (2020), Liu et al. (2015b)
Zinc (Zn)	Cu_2_O /Fe_2_O_3_	Xenon lamp	9,690	0.019	1.14% at 340 nm	NR	Pundi et al. (2025), Talibawo et al. (2022)
Copper (Cu)	TiO_2_/FeO	365nm/UVlight	UV light: ∼5,000 Visible light: ∼220	∼3	56% at 365 nm	20%–50%	Rahmawati et al. (2024), Zhang et al. (2022b)
CNT	TiO_2_/ZnO	Xenon arc lamp/AM 1.5G	12,273	2.57	NR	NR	Farah et al. (2023), Koyale et al. (2024)
Hydrogenated graphene	TiO_2_/ZnS	AM 1.5G filter	1,166	2.23	0.102%	NR	Hassan et al. (2021)
NS-rGO/RGO-MoS_2_	TiO_2_/NiCo_2_O_4_	visible light irradiation	13,996	5.36	NR	3.08	Chakrabarty et al. (2018), Chen et al. (2016)
GQDs	TiO_2_	Visible light	∼8× higher than bare TiO_2_	72	NR	0.80	Jia et al. (2022), Xiao et al. (2022)
CQDs	g-C_3_N_4_/Fe_2_O_3_	420 nm	3538.3	∼3.0	10.94% at 420 nm	NR	Hu et al. (2020)

#### 2.5.4 Graphite oxide

Graphite oxide (GO) is a good photocatalyst in hydrogen generation because of its high surface area, which is large and hydrophilic, and conduction band edge is large enough to generate the required over-potential. Although the bandgap decreases throughout the reaction, oxygenated sites that produce charge carriers are stable and readily accessible, and the hydrogen evolution is always ensured. These findings suggest that restructuring the valence band level of GO is a good strategy towards an effective overall water splitting at the irradiation of light (Yeh et al., 2010). GO in polymer form has a potential of photocatalytic evolution of H_2_ and O_2_ on water, and the band gap energy of the polymer also increases with the number of oxygen functionalities. This increase in band gap analyzed to be mainly due to downward movement of the valence band edge with the conduction band also remaining relatively constant. Accordingly, GO with adequate oxygen amounts has an electronic structure that is appropriate to enable water reduction as well as oxidation with the presence of light (Yeh et al., 2011). Although R-GO is the main absorber of UV light, CNT integration broadens the optical absorption spectrum to the visible range, which results in a higher number of electron-hole pairs. This multiphase structure enhances a photoelectrochemical activity in a significant way, almost doubling the current density of the R-GO electrode. Also, the CNTs offer high surface area, and serve as a protective layer, which guarantees stability over time and longer stability of the photoelectrode (Helmy et al., 2020).

#### 2.5.5 Quantum dots

**FIGURE 8:**
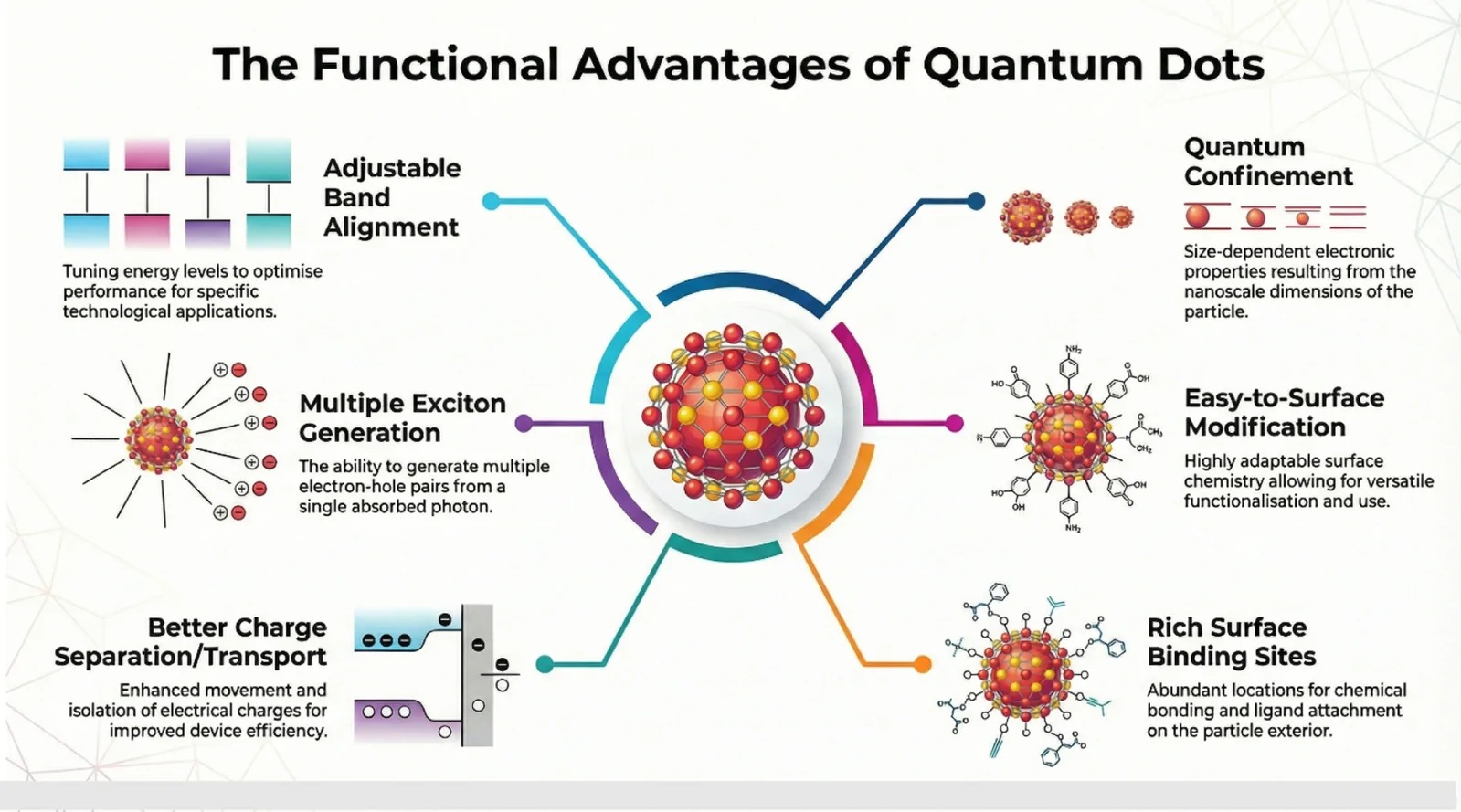
Schematic representation of the advantages of quantum dots*.* This figure was generated by the authors using Genspark and NotebookLM, and subsequently edited and annotated using Adobe Illustrator.

Carbon Quantum Dots (CQDs) also contribute greatly towards the performance of photocatalyst, by facilitating the distribution of the charges on the surface and large energy band bending, efficiently facilitates the separation of the charge carriers (Figure 8). In addition to this electronic effect, CQDs have special up-conversion characteristics, which absorb longer wavelength visible light and emit shorter wavelength light to expand light exploitation. Moreover, CQDs are efficient electron acceptors and shuttles as well, therefore, being able to transfer photo-induced electrons of semiconductor such as CdS with high speed, which reduces the rate of recombination and dramatically increases the lifetime of electron-hole pairs (Dong et al., 2021). Having a bandgap of 2.78 eV, CQDs have an excellent charge-storing capacity and serve as effective electron buffers to facilitate extraction of the conduction band. This process has a great effect on the electron-hole recombination rates. Moreover, CQDs promote electron transfer efficiency in general because they exhibit intrinsic photo-induced electron-transfer characteristics (Mehta et al., 2017).

Quantum size effects make it possible to tune the QD bandgap. Smaller CdS QDs have much higher charge transfer rates and quantum efficiency than larger ones. However, size engineering has its limits. Small QDs have stability problems, and large QDs have low charge injection efficiency. In addition, the high surface-to-volume ratio of QDs makes them sensitive to the chemical environment, which causes surface defects that trap charges. This sensitivity makes recombination centers that lower photostability and the overall performance of the device, especially when it is lit up for a long time (Jin et al., 2021). Graphene quantum dots (GQDs) make the perfect photocatalyst to hydrogen evolution as it is inexpensive, highly stable, nontoxic and its bandgap can be customized. In addition, their high oxygen affinity surface and high electron transfer capabilities enhance separation of photo-generated charges remarkably (Liang et al., 2021).

Recent progress suggests that the greatest progress in photocatalytic water splitting is now taking place at the nexus of precisely controlled active-site, compositionally-complex, and predictive electronic-structure design. Unlike conventional nanoparticle photocatalytic “cocatalysts,” which suffer from site heterogeneity and make interpretation of mechanisms challenging, single-atom photocatalysts offer atomically defined metal sites, with a maximum atom utilization ratio, and thereby allow for clear structure–activity relationships. The scientific research of high-entropy oxides can be found in the literature and they include in the lattice multiple cations that can create an unexpected defect landscape and tunable band structures that are not possible to achieve in simple binary or ternary oxides. Vacancy engineering is another method of tuning charge separation and surface reactivity by intentionally engineering and controlling oxygen or anion vacancies, which when appropriately optimized can increase visible-light absorption and carrier lifetime, but can also serve as recombination sites when over introduced. In addition to these experimental approaches, DFT-based dopant design can be predictive, enabling researchers to determine whether a dopant could produce a beneficial shallow state or deep trap state before synthesizing it, which reduces the number of experimental choices, and connects the local chemistry to the macroscopic activity. Combined these developments indicate a paradigm shift in the design of future photocatalysts from sequential material reporting to a realization of the. mechanisms involved and optimization of site identity, defect chemistry, and interfacial charge transport, while maintaining the focus on reproducibility, stability, and scalability (Li et al., 2024; Lin C. H. et al., 2024; Rezaei et al., 2026; Tsubota et al., 2025).

#### 2.5.6 Summary and future aspects

We have discussed the unique functions of Transition Metal Oxides (TMOs) and Carbon-Based Materials (CBMs) in the improved photocatalytic water splitting, in this review. The discussion shows that TMOs (e.g., Sc, V, Cr, Co) are effective in bandgap engineering, having successfully transferred the absorption spectra of host material and substrate, such as TiO_2_ and ZnO, to the visible spectrum. Such mechanisms as the creation of impurity levels and internal electric fields (e.g., in Sc-doped TiO_2_) were found to be one of the most important mechanisms towards improved light harvesting. Nevertheless, one of the drawbacks of TMOs has been the possibility of the creation of recombination centers, especially at elevated levels of doping or in oxidation states that are reactive (e.g., Cr ^6+^).

Carbon-based materials are more effective in promoting interfacial charge separation and charge-carrier transport. Their electrical conductivity is high and they are enabled to act as electron shuttles though π-conjugation, which creates Schottky junctions, which physically separate electrons and holes, reducing recombination. Although they do not play a big role in reducing the bandgap, they enhance the quantum efficiency of the reaction drastically. The upcoming investigations in the area of photocatalytic water splitting need to focus on the creation of hybrid systems that could capitalize on the benefits of both types of dopants. The critical issues encountered are optimization of dopant concentration to eliminate recombination, optimization of interface engineering to provide effective electron tunneling, and the stability problem of.

Photo-corrosion prone material such as Cu_2_O and ZnO. The subsequent generation of photocatalysts by incorporating, TMOs to absorb light and CBMs to separate the charges will have the high efficiency and stability necessary to scale up the production of green hydrogen.

Combination of data-driven catalyst discovery, scalable synthesis, durability engineering and realistic performance benchmarking will likely influence the future of photocatalytic water splitting. Recent literature reviews highlight the use of machine-learning and AI-assisted methods for screening catalysts and discovering structure–property relationships more quickly than through trial and error, as well as recent literature reviews for photocatalytic hydrogen production, which focus on the need for catalyst design, interfacing control, and system optimization for practical applications. At the same time, the field has come to realize that scalable fabrication methods are necessary to translate the materials developed at the lab scale into industrially applicable processes, especially for TMO- and CBM-based systems where the morphology, dispersion and interfacial contact play a crucial role in the performance of the material. The other big hurdle is defining realistic targets for solar-to-hydrogen efficiencies and stabilities, since many high-performing catalysts are considered sacrificial for operation or have only been tested for short periods before the test. Future development should focus on providing not only increased activity, but long-term durability, repeatability, and reactor compatibility, as these are critical for industrial application from this perspective.
